# How and When Do Leaders Influence Employees’ Well-Being? Moderated Mediation Models for Job Demands and Resources

**DOI:** 10.3389/fpsyg.2019.02788

**Published:** 2019-12-16

**Authors:** Rita Berger, Jan Philipp Czakert, Jan-Paul Leuteritz, David Leiva

**Affiliations:** ^1^Department of Social Psychology and Quantitative Psychology, Facultat de Psicologia, Universitat de Barcelona, Barcelona, Spain; ^2^Human Factors Engineering, Fraunhofer Institute for Industrial Engineering (IAO), Stuttgart, Germany

**Keywords:** job demands-resources model, transformational leadership, passive-avoidant leadership, role ambiguity, team climate for learning, job autonomy, employee well-being

## Abstract

Following the call of recent reviews on leadership and well-being, the purpose of this study is to examine how and when two contrasting leadership styles, transformational leadership (TFL) and passive-avoidant leadership (PAL), are related to employees’ anxiety and thereby either promote or inhibit employees’ well-being. Using the prominent job demands-resources (JD-R) model as a theoretical framework, we propose that the relationship between leadership behavior and anxiety is mediated by organizational job demands, namely, role ambiguity (RA), and job resources, namely, team climate for learning (TCL), as well as moderated by autonomy as important job characteristic. A sample of 501 knowledge workers, working in teams in a German research and development (R&D) organization, answered an online survey. We tested moderated multiple mediation models using structural equation modeling (SEM). Results demonstrated that the relationships between TFL as well as PAL on the one hand and anxiety on the other hand were fully mediated by RA and TCL. Job autonomy moderated the quality of the leadership–job demand relationship for TFL and PAL. This paper contributes to understanding the complex relationship between leadership and followers’ well-being taking into account a combination of mediating and moderating job demands and resources. This is the first study that examines the effects of TFL and PAL on well-being taking into account the job demand RA and team processes and autonomy as resources.

## Introduction

Modern business organizations are increasingly aware of the importance to sustain and promote employees’ well-being in order to gain and maintain competitive advantage (Nielsen et al., 2017). Simultaneously, the fast-paced and increasingly complex environments in which organizations operate demand for constant innovation (Reuveni and Vashdi, 2015) and performance (Nielsen et al., 2017). Consequently, teamwork and collaborative learning are becoming more important, since teams pool different skills and expertise to deal with new and diverse tasks (Edmondson and Nembhard, 2009; Ramírez Heller et al., 2014). Additionally, promoting teamwork in organizations is often seen as a means to attain organizational goals and build competitive advantage (Day et al., 2004). Teams also have many resources that have been associated with higher levels of well-being and lower strain (Avanzi et al., 2015), explaining why working in teams has also been linked to employee well-being (e.g., Di Fabio, 2017).

Albeit organizational and psychological benefits of working in a team, also psychosocial risk factors may arise which may lead to employee stress (Navarro et al., 2011). For example, it is a basic tenet in psychological science that stress originates from dealing with ambiguity and uncertainty (Peters et al., 2017). This is especially applicable to knowledge workers that are exposed to uncertain tasks, tough deadlines, and conflicting requirements (e.g., Savelsbergh et al., 2012; Leuteritz et al., 2017). Consequently, organizations need to prepare their employees successfully to deal with ambiguity and provide the necessary resources for well-being. In this regard, collaborative learning is thought to help in dealing with those challenges and acts as an interpersonal resource (Lases et al., 2019).

Another way of dealing with these ambiguity-related work demands is transformational leadership (TFL) behavior, as it offers support resources via, e.g., individual consideration and has been recognized since several years ago as important for employees’ well-being (Kelloway and Barling, 2010; Arnold, 2017). Over the last few years, researchers’ interest in the relationship between TFL (Bass, 1985) on employee well-being has grown, and the amount of available literature has increased substantially (Skakon et al., 2010; Arnold, 2017). Despite the fact that the relationship between TFL and employee well-being has been well-established in research, the exact underlying complex mechanisms through which transformational leaders exert their health-promoting effects still remain unclear (Skakon et al., 2010; Arnold, 2017). Specifically, the questions of how and when leadership affects followers’ well-being, and even more interestingly how these mechanisms can be combined in models, are yet to be addressed (Arnold, 2017).

Our study aimed to invoke answers to these questions following the suggestions of the recent reviews (Arnold, 2017; Inceoglu et al., 2018) by exploring possible both, mediating and moderating mechanisms between TFL and employees’ well-being. Furthermore, previous research lacked a clear theoretical approach regarding the inclusion or not of specific mediators in the TFL–well-being relationship (Diebig et al., 2017). Our study, in line with recent relevant work (e.g., Schaufeli, 2015; Diebig et al., 2017), tried to overcome this limitation by using the job demands-resources (JD-R) model (Bakker and Demerouti, 2007) as a prominent theoretical framework, which allowed us to combine job demands and job resources. As leadership behavior does not always affect followers’ well-being in the same way (Inceoglu et al., 2018), we decided to analyze differential mediator pathways including two different leadership behaviors and two different mediators, specifically job demands and resources that should facilitate information about how leadership behavior increases resources and decreases demands and impacts in followers’ well-being. We therefore used TFL, a change-oriented leadership style with a positive relationship to well-being (Inceoglu et al., 2018), and passive-avoidant leadership (PAL) (Heinitz et al., 2005), a passive leadership style with a negative relationship to well-being (Frooman et al., 2012); the latter has been researched less (Inceoglu et al., 2018). Furthermore, the selection of our mediators was based on Job design (Hackman and Oldham, 1976) and included the job demand and work characteristic role ambiguity (RA) and therefore a motivational mediator. Additionally, our mediator selection was based on the social learning theory (Bandura, 1977), including the job resource of team climate for learning (TCL) (Brodbeck et al., 2010), a relational and social-cognitive mediator, since both pathways are relevant for both positive and negative leadership styles (Inceoglu et al., 2018). We selected the concept of RA as one of the most commonly investigated occupational stressors (Bowling and Beehr, 2006), related to negative well-being outcomes, and TCL, which is a relevant resource for well-being (Lases et al., 2019). In this sense, TFL is known to positively influence employee outcomes via, e.g., inspirational motivation, which is in line with the motivational path of the JD-R and which has also been shown to increase learning resources of employees via, e.g., intellectual stimulation (Schaufeli, 2015). Contrastingly, PAL does not increase resources (Barling and Frone, 2017). We chose state anxiety to be an important negative aspect of subjective well-being (Rajgopal, 2010; Skakon et al., 2010; Cheng and McCarthy, 2018). It describes the proximal and internally directed response to perceived stressors (Glazer and Kruse, 2008; Pyc et al., 2016) and is known to be positively related to RA (Glazer and Beehr, 2005). Thus, we contributed to research on negative indicators of well-being which until now have been less researched in relation with leadership behaviors (Inceoglu et al., 2018) but which are necessary to understand the mechanisms included in the negative pathway. Until now, few studies (e.g., Cole et al., 2009; Franke and Felfe, 2011; Holstad et al., 2014; Diebig et al., 2017) investigated the role of moderators for the TFL–followers’ well-being relationship showing the importance of the conditions when TFL predicts employees’ well-being. Less studies also investigating moderating effects in mediation models (Holstad et al., 2014; Diebig et al., 2017) with mixed results. Hence, we followed a recent call (Arnold, 2017) that requires further research on moderated mediation to answer how and when leadership influences followers’ well-being. Therefore, we integrated with job autonomy one well-known moderator, a core job resource in line with the JD-R model (Bakker and Demerouti, 2007) that describes a motivational aspect (Inceoglu et al., 2018). More specifically, to answer the question of how and when leadership influences employee well-being, we propose that the relationship between TFL and anxiety is mediated by RA and TCL, and that the quality of the TFL -RA relationship is influenced by job autonomy. As research on negative leadership style and employees’ well-being and the underlying mechanism is scare and needed (Inceoglu et al., 2018), we include PAL as “contrasting” to TFL (Hetland et al., 2011, p. 163) to uncover and evidence the linkages of both sides of the leadership spectrum with job demands and resources, and ultimately with followers’ well-being. In line with this, we propose that the relationship for TFL’s negative counterpart, namely, PAL, is also mediated and moderated by the same factors. This is in line with previous studies that mirrored the effects of PAL and TFL in one study to highlight the importance of TFL and the destructiveness of PAL (e.g., Hetland et al., 2011; Frooman et al., 2012) via the same mechanism.

With this study, we contribute to existing research in several ways. First, we further built up on the literature combining the JD-R Model and the TFL employee well-being relationship. To our knowledge, relevant research combining both, job demands and job resources in the same model has been limited (Hentrich et al., 2017). Thus, by analyzing the role of leadership together with motivational and social-cognitive and relational mechanisms (Inceoglu et al., 2018), as well as influencing core job characteristics, we tried to overcome limitations of previous research. Specifically, we broadened the scope of mediators of the TFL–well-being relationship by including TCL and RA. Second, we highlighted influencing job characteristics as moderators in the TFL–well-being relationship. Third, we mirrored the effect of TFL with the antagonist PAL, so that the contrast of the leader’s effect becomes even more apparent and, in this manner, we increase the scarce research on this topic. Lastly, considering that research in TFL and well-being has shown inconsistencies in regard of whether the relationship between TFL and well-being is fully or partially mediated (Arnold, 2017), we explored both, direct and indirect effects and thus, contribute to fill this research gap.

## Theoretical Background and Research Model

The JD-R model is the most prominent framework to study the effects of variables on different levels (structural, interpersonal, etc.) on employees’ mental health (Nielsen et al., 2017). Compared to conservation of resources theory (Hobfoll and Lilly, 1993; Halbersleben et al., 2014), which also underlines the importance for the individual to gain and preservation of resources, the JD-R model offers an appropriate consideration of job demands with respect to the available resources, without narrowing its focus on resources acquisition and conservation.

According to the JD-R model, every working environment has its own specific factors linked to job related stress. These factors can generally be classified into two categories: job demands and job resources. Job demands “refer to those physical, psychological, social, or organizational aspects of the job that require sustained physical and/or psychological (cognitive and emotional) effort or skills and are therefore associated with certain physiological and/or psychological costs” (Bakker and Demerouti, 2007, 312), while job resources “refer to those physical, psychological, social, or organizational aspects of the job that are either/or functional in achieving work goals, reduce job demands and the associated physiological and psychological costs, and stimulate personal growth, learning, and development” (Bakker and Demerouti, 2007, 312). Within the JD-R as a broad framework, diverse pathways (Inceoglu et al., 2018) can be combined. Building on job design (Hackman and Oldham, 1976), motivational pathways using motivational mediators and demands allow to analyze organizational information that can lead to redesign. Grounding on the social learning theory (Bandura, 1977) for social-cognitive and relational pathways using this category of mediators and resources allow to analyze developmental aspects that can lead to development.

### The JD-R Model and Leadership

There is a growing number of studies that show the link between leadership style and employee well-being (e.g., Kelloway and Barling, 2010) but that often lack a clear conceptual approach (Inceoglu et al., 2018). The JD-R model offers a broad theoretical framework for explaining the indirect positive relationship between TFL and well-being (Diebig et al., 2017) and a negative one with PAL (Molero Alonso et al., 2010). Being initially included as a job resource (Bakker and Demerouti, 2007), recent research integrated leadership as a standalone predictive factor to the JD-R that affects both, job demands and resources (e.g., Schaufeli, 2015; Diebig et al., 2017). This is in line with the social environment model (Katz and Kahn, 1978), which suggests that perceptions of a leader frame a person’s perceptions of experienced work characteristics, which in turn relate to outcomes and strains. Thus, it is widely regarded as a contextual variable (Skakon et al., 2010; Oc, 2018). Therefore, we contributed with our study in the frame of the prominent JD-R with a concept-based analysis approach. To be able to get differential information on the mechanisms working in the leadership–well-being relationship, we included two different leadership styles in the analysis: the positive and change-oriented TFL and the negative and passive PAL that until now has been researched less (Arnold, 2017; Inceoglu et al., 2018).

### Transformational Leadership and Employee Well-Being

Transformational leadership, based on the full range leadership model (Bass, 1985), is one of the most widely used leadership theories (Gawke et al., 2017) and contributes to the personal growth of followers by providing them with idealized influence, individualized consideration, intellectual stimulation, and inspirational motivation (Bass, 1990, 2000). It is related to multiple positive outcomes at individual and team level (Kelloway et al., 2012; Harms et al., 2017) and considered as change-oriented leadership (Inceoglu et al., 2018). Since recently, TFL is recognized as a leadership style that as an antecedent positively influences employee well-being (Arnold, 2017), such as decreased risk of sick absence (Kuoppala et al., 2008; Skakon et al., 2010), and that reduces stress (Harms et al., 2017). This has been well documented in both, profit and non-profit organizations (e.g., McMurray et al., 2010) as well as in cross-sectional (e.g., Arnold et al., 2007) and longitudinal studies (e.g., Tafvelin et al., 2011). According to the JD-R model, leaders manage the allocation of job demands and job resources on their followers (Schaufeli, 2015). In other words, TFL leaders provide job resources, strengthen subordinates’ personal resources and reduce job demands (Diebig et al., 2017; Hentrich et al., 2017). For example, leaders may provide social support (Holstad et al., 2014) through individualized consideration or reduce job demands if one cannot adequately carry out the responsibilities (Holstad et al., 2014; Hentrich et al., 2017). Also, transformational leaders who encourage subordinates to use resources effectively for coping are a beneficial resource for them (Harms et al., 2017). Thus, they promote workers’ well-being through healthy practices at work (Inceoglu et al., 2018).

However, the underlying mechanism behind the relationship between TFL and various indicators of well-being is still unclear (Skakon et al., 2010; Arnold, 2017). Research on TFL and employee well-being has thus proposed models where job demands or job resources mediate the TFL well-being relationship (Holstad et al., 2014; Schaufeli, 2015; Diebig et al., 2017; Hentrich et al., 2017). Nevertheless, research on how transformational leaders promote follower well-being that includes both, job demands, and job resources at the same time has clearly received less attention (Hentrich et al., 2017). For example, Hentrich et al. (2017) found that TFL is associated with lower job demands, such as work intensity, but higher occupational self-efficacy (personal resource), which in turn contributes to lower levels of strain (here irritation). Similarly, it has been found that daily TFL behavior is related to followers’ daily level of stress on a day-to-day basis through role conflict (job demand) and team cooperation (job resource) (Diebig et al., 2017). To broaden the existing evidence base (Diebig et al., 2017; Hentrich et al., 2017), the combined analysis of job demand and resources will be one of the contributions of our paper.

### Passive-Avoidant Leadership and Employee Well-Being

Based on the review done by Inceoglu et al. (2018), additional information on how leadership influences employee well-being is needed. To underscore the importance of TFL, to better understand its relationship with well-being and to contribute to closing the apparent research gap regarding passive forms of destructive leadership styles (Inceoglu et al., 2018), this study also included PAL as an independent variable (Frooman et al., 2012; Barling and Frone, 2017).

In the contrast to the active form of TFL, other authors have linked management by exception passive, previously embedded in the transactional style (Bass, 1998; Avolio, 1999; as cited in Judge and Piccolo, 2004) of the full range leadership model (Bass, 1985), and laissez-faire style under the label of PAL (Heinitz et al., 2005). PAL represents both the passive mode of reaction and the lack of reaction at all (Molero Alonso et al., 2010).

Passive-avoidant leadership-related behaviors do not accomplish any of the tasks associated with TFL. Working with leaders that do not provide subordinates with relevant feedback, employees are less likely to know how they have to orientate their efforts to complete their task. Not only is it argued that employees under PAL lack social support by the supervisor, it is also assumed resources are not strengthened, and job demands such as role stressors can even increase, consequently leading to increased health impairment (Barling and Frone, 2017). Research on PAL is scarce (Chênevert et al., 2013; Barling and Frone, 2017). To the authors’ knowledge, no comprehensive meta-analyses on the specific issue exist until today. Nonetheless, some articles provided strong empirical support to link passive leadership styles to decreased employees’ well-being. For instance, some authors (Skogstad et al., 2007) have found a positive relationship between laissez-faire leadership (embedded in PAL) on the one hand, and role conflict and RA on the other hand. Furthermore, they have also found the effect of laissez-faire on distress (as well as on workplace bullying) to be mediated by these stressors. Moreover, such a relationship has also been found between passive-avoidant leaders and the following variables: role overload, role conflict, and RA (Chênevert et al., 2013; Barling and Frone, 2017). Furthermore, we follow the idea of Frooman et al. (2012), who argued earlier that PAL could be seen as the very opposite of TFL. Specifically, they state that “by their very definitions, it does not seem possible for someone to be both transformational and passive avoidant” (Frooman et al., 2012, p. 450). In this sense, it seems reasonable to argue that when PAL prevails, all the positive effects of TFL on employees’ well-being are lacking. For instance, within the same research, it was shown that TFL positively predicts job satisfaction of the follower, whereas PAL negatively predicts the same factor of well-being (Frooman et al., 2012). Following the conclusion by Inceoglu et al. (2018), this study contributed to a better understanding of how the positive change-oriented TFL influences followers’ well-being by including the negative passive PAL into the analysis.

### Anxiety as a Negative Indicator of Well-Being

We chose state anxiety as an important negative well-being indicator as outcome variable (Rajgopal, 2010) for several reasons. First, rather few studies analyze the relationship between leadership and negative indicators of employees’ well-being, e.g., anxiety (Montano et al., 2017; Inceoglu et al., 2018). Second, it is defined as “a state physio-psychological sensation, addressing people’s perceptions of psychological and physiological states” (Glazer and Beehr, 2005, 469) and includes both, physiological and psychological outcomes of stress. Third, anxiety is especially related to RA across different countries (Glazer and Beehr, 2005) in working environments that are characterized by uncertainty and ambiguity (Leuteritz et al., 2017; Peters et al., 2017). Fourth, it has also been conceptualized as the most proximal response in supervisor–subordinate relationships that is directed internally (Pyc et al., 2016). Fifth, we analyzed state anxiety as a crucial outcome because it is a great indicator for one of the most frequent mental disorders, namely, anxiety disorder, in the European Union (Wittchen et al., 2011). Sixth, when dealing with work-related stress, state anxiety is seen as an initial response to stress (Glazer and Kruse, 2008) and significantly related to the outcome of chronic work-related stress (Koutsimani et al., 2019), organizational commitment, and turnover intention (Glazer and Beehr, 2005). So, it makes sense to attack the predictor of those later consequences in an early stage to avoid severely negative outcomes.

### Mediators

To get information on and contribute to answering the question *how* leadership behavior influences followers’ well-being, we included two different mediators in the leadership well-being relationship. Within the broad JDR, we combine and base on job design (Hackman and Oldham, 1976) for demands and social learning theory (Bandura, 1977) for resources two theoretical approaches that can allow following Inceoglu et al. (2018) to analyze two important mediator pathways: a motivational one via the demand RA and a social-cognitive and relational one via resource TCL.

#### Role Ambiguity as a Job Demand

Some researchers have analyzed work characteristics as mediators in the relationship between TFL and followers’ well-being. For example, Nielsen et al. (2008) found in a longitudinal study that work characteristics partially mediated the relationship between TFL and followers’ well-being at time 1 and fully mediated the same relationship at time 2. Even so, there is not yet enough research suggesting that leaders may reduce strain via job demands (Hentrich et al., 2017).

Building on Job design (Hackman and Oldham, 1976), we have chosen with RA, which is one of the most investigated occupational stressors (Bowling and Beehr, 2006), an important and well-known work characteristic and demand. RA, which has been linked with multiple indicators of strain, e.g., the frequency of symptoms of depression, generalized anxiety, hyper-alertness (Dobreva-Martinova et al., 2002), and tension/anxiety (Parker and DeCotiis, 1983; Glazer and Beehr, 2005), is prevalent in research organizations (Schulz, 2013), and thus, relevant to our sample that consisted of employees of a research organization. Those knowledge workers are often confronted with uncertain tasks, conflicting priorities, multiple stakeholders, and requirements that may exceed their available resources (Savelsbergh et al., 2012; Leuteritz et al., 2017). At the same time, teams often suffer compositional changes that provoke a change in responsibilities which may, in turn, result as unclear or incompatible (Alcover et al., 2011). RA constitutes a demotivational job design (Hackman and Oldham, 1976; Inceoglu et al., 2018). RA refers to feelings of uncertainty or to a lack of necessary information concerning role functions and responsibilities (Glazer and Beehr, 2005; Örtqvist and Wincent, 2006). Previous research has linked role stressors to the JD-R (Bakker and Demerouti, 2007; Hentrich et al., 2017) and showed that transformational leaders may actively play a role in decreasing RA and consequently, reducing anxiety (e.g., Diebig et al., 2016). This may be done by clarifying roles and motivating team members by providing a vision. Moreover, transformational leaders may train and stimulate employees to be able to conduct special non-routine decision-making (Harms et al., 2017). In contrast, PAL has been associated with perceptions of unclear guidelines at work (Hetland et al., 2011), demonstrating that leaders who neglect their duties create uncertain and ambiguous environments. Other studies link PAL particularly to RA (Skogstad et al., 2007; Chênevert et al., 2013; Barling and Frone, 2017). In the present study, we included RA as a job demand and motivational mediator to be able to analyze how both leadership styles are influencing the well-being of followers. This contributes to the scarce existing research.

H1a: RA mediates the negative relationship between TFL and anxiety.

H1b: RA mediates the positive relationship between PAL and anxiety.

#### Team Climate for Learning as a Job Resource

Grounding on social learning theory (Bandura, 1977), the TFL (Inceoglu et al., 2018) has also been related to diverse team processes as resources such as team cooperation (Diebig et al., 2017). This resource that, e.g., offers social support (Bakker and Demerouti, 2007), therefore, may be also linked to reduced strain (Schaufeli, 2017). Some researchers found team self-efficacy (Nielsen et al., 2009) or a positive climate for innovation (Tafvelin et al., 2011) mediating the relationship between TFL and employees’ well-being. As a recent study could show, the aspect of having a positive learning climate is crucial for the individual well-being of the team members (Lases et al., 2019).

Nevertheless, there is not yet much research that analyzes the role of mediating team processes in the leadership–employee well-being relationship (Jung and Sosik, 2002), and even less applying TCL as a job resource. TCL is defined as “shared individual perceptions of work settings among members of a team or an organization that promote or hinder learning in the workplace” (Ramírez Heller et al., 2014, p. 544). Team members with positive TCL (Brodbeck et al., 2010): (1) offer support to the other members and show understanding and mutual trust; (2) have regular contact with formal or informal communication; (3) work toward common goals with clear, realistic, and achievable objectives while a notion of democracy prevails with no team member dominating over others; and (4) perceive a kind of individual development as a result of the group enhancing their creativity and providing them with various resources such as new ideas and support.

Based on the JD-R, in the present study, TCL is considered therefore as a job resource and relational and social-cognitive mediator (Inceoglu et al., 2018) in the leadership–well-being relationship for various reasons: it may reduce job demands (e.g., Lases et al., 2019), it promotes individual development and resources, since it stimulates learning, and it is functional in achieving work goals, since it is linked to increased positive performance indicators, team functioning, and support for innovation (Ramírez Heller et al., 2014). Moreover, according to Bakker and Demerouti (2007), job resources may include opportunities for development, feedback, and role clarity, while daily team cooperation is a job resource that is linked to reduced daily levels of stress (Diebig et al., 2017). As described above, a positive TCL facilitates individual development while mutual trust and face-to-face frequent contact may help team members in clarifying role expectations (Marín Puchades, 2015). It can be concluded that working in a team that is prevailed by a positive climate for learning can make employees less vulnerable to resource loss and more capable and likely to gain other resources, which, in turn makes them more resilient to stress (Hobfoll and Lilly, 1993; Xanthopoulou et al., 2009). This possibly promotes the growth of personal competences of employees, making them more efficient in dealing with demands, and may influence in the reduction of stress reactions (Dackert, 2010). Previous research linked TFL and PAL with the learning climate in a team setting and emphasized both the constructive impact of TFL and destructive impact of PAL on the learning climate (e.g., Hetland et al., 2011). According to the input-mediator-output (IMO) model (Ilgen et al., 2005), team climate has been widely regarded as an emergent state (Mathieu et al., 2008). TFL may be considered as an input variable which affects TCL which, in turn, may affect team members’ well-being, e.g., anxiety levels.

H2a: TCL mediates the negative relationship between TFL and anxiety.

H2b: TCL mediates the positive relationship between PAL and anxiety.

### Job Autonomy as an Interacting Factor in the Leadership–Job Demands Relationship

To get information about when leadership behavior influences followers’ well-being, we wish to add to the present lack of research (2017) and included with job autonomy an important motivational moderator, since it seems to be one of the most powerful job resources (Bakker and Demerouti, 2007) and simultaneously an influencing factor for TFL leadership effectiveness (Den Hartog and Belschak, 2012).

Job autonomy is defined as “the degree to which the job provides substantial freedom, independence, and discretion to the individual in scheduling the work and in determining the procedures to be used in carrying it out” (Hackman and Oldham, 1976, p. 258). It constitutes a contextual variable, where high levels of autonomy describe contexts in which employees can take full advantage of their abilities at the job (Morgeson et al., 2005), and where environments are more dynamic and uncertain (Den Hartog and Belschak, 2012). There is strong evidence that our sample of knowledge workers in research and development (R&D) organizations work in such described environments (Schulz, 2013).

Former research suggested that certain leadership theories, such as leader–member exchange (Volmer et al., 2012), empowering leadership (Ju et al., 2019), and also TFL (Den Hartog and Belschak, 2012), work better in less-prescribed environments by modeling successfully the moderating function of job autonomy in their works. It was found that leaders through work characteristics such as, e.g., autonomy might enable resources (Halbersleben et al., 2014). In line with this, we argue that the impact of leadership on the job demand RA is influenced by this core job characteristic (Hackman and Oldham, 1976), and that this applies for both contrasting leadership styles, TFL and PAL. These assumptions seem legitimate for the following reasons:

First, the link of TFL on RA arguably depends strongly on the actual capabilities of the employee to clarify his or her working situation (Volmer et al., 2012; Ju et al., 2019). For instance, when employees are receiving motivational triggers by the transformational leader to schedule their own action plans and to clarify their roles, they are likely to do so if the job context allows it (Ju et al., 2019). In contrast, when job autonomy is low, the leader might stimulate employees to enact clarifying activities, albeit the role stressor will continue to prevail because the specific working context is determined (Volmer et al., 2012). Hence, we expect that the TFL–RA relationship is influenced by job autonomy, and specifically that the higher the job autonomy level, the greater the link of TFL.

Second, the reinforcing effect of PAL on RA is arguably influenced by the degree of autonomy in the way that PAL paired with a low level of autonomy strengthens the effect of a leader’s passive-avoidant behavior on RA. When employees are not able to reduce ambiguity by themselves, or autonomously, the perception of ambiguity due to leader passivity might be even higher (Hetland et al., 2011).

H3a: Job autonomy moderates the relationship between TFL and anxiety via RA. The higher the levels of job autonomy, the higher the influence of TFL on RA.

H3b: Job autonomy moderates the relationship between PAL and anxiety via RA. The lower the levels of job autonomy, the higher the influence of PAL on RA.

**FIGURE 1:**
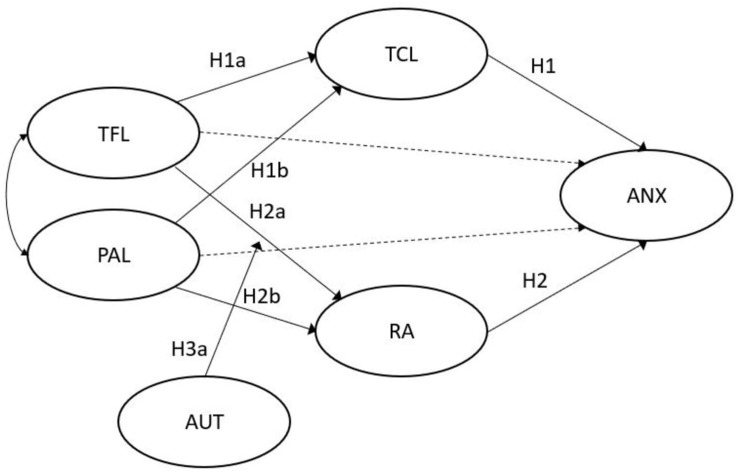
Hypothesized model TFL.

**FIGURE 2:**
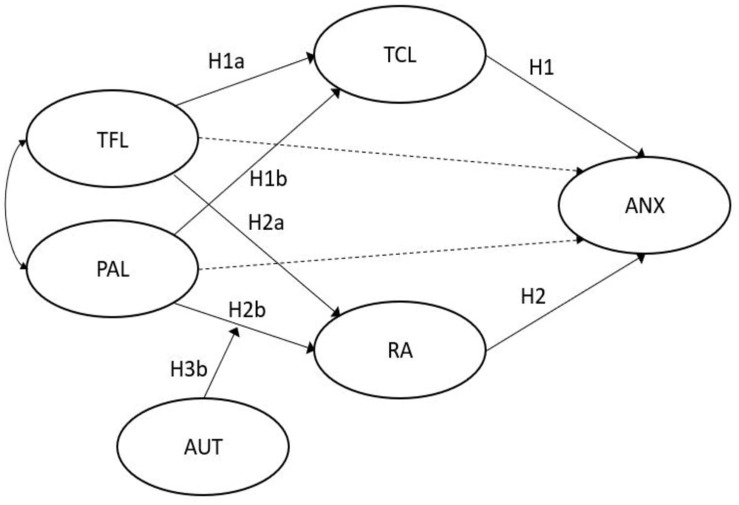
Hypothesized model PAL.

In conclusion, we hypothesize moderated multiple mediation models where RA and TCL mediate the relationship between TFL and strain. We argue that TFL is positively related to TCL and in turn negatively related to anxiety. Similarly, we hypothesize that TFL is negatively related to RA and in turn, negatively related to anxiety. Job autonomy moderates the effect (Figure 1). In contrast, we further hypothesize that PAL is negatively related to TCL and positively related to RA, which eventually increases anxiety. Job autonomy is hypothesized to moderate the effect between PAL and RA (Figure 2).

## Materials and Methods

### Participants and Procedure

A total of 501 employees of an R&D organization of approximately 15,000 employees participated in an online survey in Germany. As Table 1 shows, out of the 501 participants, 343 (68.5%) were males and 158 (31.5%) were females with ages ranging from 18 to 70 years (*M* = 35.6, *SD* = 10). Moreover, all participants were part of teams, mainly R&D teams (85.8%). A lower percentage of individuals participated in administration (9.6%), facility (2%), and other tasks (2.6%).

**TABLE 1:** Sample description (*N* = 501).

	***n***	**Percent (%)**
Male participants	343	68.5
Female participants	158	31.5
Job: R&D	430	85.8
Job: administration	48	9.6
Job: facility	10	2
Job: other	13	2.6

In order to measure the constructs involved in this research, four questionnaires were applied with a total of 48 items, as well as questions regarding demographic data. All scales used where translated into German following the guidelines of the International Test Commission (2017) for translating and adapting tests. All team leaders were excluded from the data collection, and all participants were informed about data confidentiality policy in regard of the administration of the questionnaire by a written text. Moreover, participation was voluntary; the declaration of consent of the individuals was asked before completion of the questionnaire and found to be accepted when submitted. In order to promote participation, a lottery of vouchers was used.

There were no empty cells in the final dataset, as participants could only return completely answered questionnaires. Hence, missing values were not an issue in this study.

### Measures

#### Transformational Leadership

We measured TFL, based on the full range leadership model (Bass, 1985), using the German version of the Human System Audit Short-Scale of TFL shown to be unidimensional (Berger and Antonioli, 2019), since it is shorter than the Multifactor Leadership Questionnaire MLQ-5X-Short (Avolio and Bass, 2004) and an easy to apply instrument (Berger et al., 2011). The scale consists of eight items (e.g., “My leader promotes the use of intelligence a means of overcoming obstacles”) that measure TFL using a five-point Likert scale (from 1 = *strongly disagree* to 5 = *strongly agree*). Previous research has provided empirical evidence for the construct validity of this measure (Berger et al., 2011, 2012) in diverse languages. Cronbach’s alpha for the eight items for the German sample was α = 0.93.

#### Passive-Avoidant Leadership

For PAL (Heinitz et al., 2005), we used the Multifactor Leadership Questionnaire MLQ-5X-Short (Avolio and Bass, 2004). The answers to the items are measured with a five-point Likert scale with responses regarding frequency (from 1 = *never* to 5 = *nearly always*). We only used the eight items that measure PAL. Four of these items belong to the management by exception passive sub-scale (e.g., “Avoids intervening until the problems get serious”) and four represent laissez-faire style (e.g., “Has avoided telling me how to perform my job”). Cronbach’s alpha for the PAL was α = 0.88, which implies a high reliability. This is in line with previous studies (e.g., α = 0.84 in Molero Alonso et al., 2010).

#### Role Ambiguity

Three items of The Role Stressors Questionnaire (Glazer and Beehr, 2005) that refer to RA (e.g., “I know exactly what is expected of me”) were translated into German and used to measure RA following the ITC Guidelines (2017). The items are rated with a seven-point Likert scale (from 1 = *strongly disagree* to 7 = *strongly agree*). The Cronbach’s alpha in our sample for the three items used was α = 0.81.

#### Team Climate for Learning

The German version of the TCL Questionnaire (Brodbeck et al., 2010) was used for measuring TCL. Thirty-three items were used that include nine subscales: (1) Mutual Trust, (2) Goal Alignment, (3) Attendance, (4) Regular Contact, (5) Democracy, (6) Team Management, (7) Individual Development, (8) Open Exchange, and (9) Motivation and Interest (Brodbeck et al., 2010; Ramírez Heller et al., 2014). The items (e.g., “My team provides me with useful ideas and practical support”) were rated using a seven-point Likert scale (from 1 = *strongly disagree* to 7 = *strongly agree*).

#### Job Autonomy

Three items of the German version of the well-established Job Diagnostics Survey (Hackman and Oldham, 1976; Schmidt et al., 1985) were used to assess job autonomy. One item was “How much autonomy is there in your job? That is, to what extent does your job permit you to decide on your own how to go about doing the work?” and is rated with a seven-point Likert scale (from 1 = *very little; the job gives me almost no personal “say” about how and when the work is done* to 7 = *very much; the job gives me almost complete responsibility for deciding how and when the work is done*). The other two items are “*The job gives me considerable opportunity for independence and freedom in how I do the work*” and “*The job denies any chance to use my personal initiative or judgment in carrying out the work.”* These items are rated with a seven-point Likert scale (from 1 = *strongly disagree* to 7 = *strongly agree*). Cronbach’s alpha in our sample for the three items used was α = 0.77.

#### Strain

We translated the four items out of the Anxiety Scale of Parker and DeCotiis (1983) that refer to anxiety (e.g., “I have felt fidgety or nervous as a result of my job”) into German. Similar to previous research, the remaining 11 items of the scale were omitted because they were referring to time stress (Glazer and Beehr, 2005). The participants were asked to answer the questions using a seven-point Likert scale (from 1 = *strongly disagree* to 7 = *strongly agree*). Cronbach’s alpha in our sample for the four items used was α = 0.88.

### Data Analysis

Our hypothesized models, displayed in Figures 1, 2, are moderated multiple mediation models where TFL and PAL are the independent variables, anxiety the dependent variable, RA and TCL two mediators, and job autonomy the moderator in the leadership–job demand relationship. In order to test the hypothesized models, we applied structural equation modeling (SEM) using IBM SPSS AMOS 25.0 (Arbuckle, 2017). We assessed the hypothesized moderating effects separately, because we were interested in the specific interaction effects for the individual leadership style (Dawson, 2014).

To assess the impact of common source variance, we followed the recommendations of experts in the field (Podsakoff et al., 2003, 2012) and applied the so-called “single-common-method-factor approach” (Podsakoff et al., 2003, 895) with CFA. Hence, we included an additional latent variable to our CFA model and allowed all items to load on their theoretical constructs as well as on a common method variance factor. Subsequently, the common method variance factor was removed, and the difference of the loadings of the indicators on their constructs between the models was compared. Since standardized cut-off criteria for this method are yet to be agreed on, we selected differences higher than 0.2 as our criterion based on Cohen’s (1988) effect size categorization. For hypothesis testing, we set the significance level at α = 0.05.

In order to validate the questionnaire for TCL, we split the sample in half and carried out an exploratory factor analysis (EFA) with one half, followed by a confirmatory factor analysis (CFA) (Cabrera-Nguyen, 2010). EFA revealed a four-factor structure for 33 items. The four factors were labeled as: (1) Group Interaction and Group Support (e.g., “My team provides me with useful ideas and practical support”), (2) Goal Alignment (e.g., “The goals of my group are useful and suitable”), (3) Open Exchange (e.g., “All opinions are respected”), and (4) Regular Contact (e.g., “The group members meet frequently to discuss formal and informal topics”). Factors 1–3 showed a very good internal consistency with alphas ranging from α = 0.89 to α = 0.92. Cronbach’s alpha for factor 4 was α = 0.64.

This four-factor solution was supported by using a second-order CFA. The CFA revealed a satisfactory model fit [χ^2^_(__185__)_ (*N* = 247) = 532.61, *p* < 0.001; χ^2^/*df* = 2.879; Tucker–Lewis Index (TLI) = 0.89; comparative–fit index (CFI) = 0.91; root-mean-square error of approximation (RMSEA) = 0.087]. Values larger than 0.90 for TLI and CFI and lower than 0.10 for RMSEA indicate an acceptable model fit (Byrne, 2009). Regarding the χ^2^/df, various rules of thumb have been recommended; a value up to 2.0 is considered very good and between 2.0 and 5.0 acceptable (Hair et al., 2006). All of the standardized parameter estimates (i.e., factor loadings) were significant at *p* < 0.01 and ranged from 0.51 to 0.97 (mean standardized loading = 0.78).

Maximum likelihood estimation was applied and the goodness-of-fit of the tested models was evaluated using several indices: (1) the chi-square test statistic, (2) the normed chi-square (χ^2^/*df*), (3) the RMSEA, (4) the TLI, and (5) the CFI. Based on recommendations by Hu and Bentler (1998), the following cut-offs were used to indicate adequate model fit: TLI and CFI > 0.95 and RMSEA < 0.06.

The tests of indirect effects (i.e., Hypotheses 1 and 2) were analyzed simultaneously, and a two-step approach was adopted (Kline, 2016). First, a measurement model was tested. This model determines if the indicators well reflect the latent constructs. Finally, the second step (structural part) allows us to test the proposed relationships among the latent variables. As proposed by Preacher and Hayes (2008), we applied a bootstrap procedure for testing statistical significance of mediations. More specifically, 1000 bootstrap samples were produced which were used to estimate 95% bias-corrected confidence intervals (CIs) to test for significant indirect effects.

To test the interaction effect of leadership and job autonomy on RA, we adopted the three-step procedure of residual mean centering for latent constructs interaction effects introduced by Little et al. (2006), since – unlike mean centering – it completely alters item variances and covariances and is therefore a recommended orthogonalizing tool for addressing interaction effects in SEM (Geldhof et al., 2013). In a first step, all first-order variables of the two latent variables were multiplied. In a second step, each product term was regressed on all effect indicators. Finally, the resulting residuals were integrated in the model and the covariances between the two latent variables and the interaction effect were set to zero. Likewise, 1000 bootstrap samples were produced, and 95% CI analyzed to test the interaction effect for significance (Little et al., 2006).

## Results

### Preliminary Analysis

Table 2 displays the means, standard deviations, Cronbach’s α, and intercorrelations of all variables included in this study. All measures relevant to our proposed mediation model were significantly correlated; in other words, measures of TFL, PAL, the hypothesized mediating variables (TCL, RA), the hypothesized moderator job autonomy, and anxiety were correlated as expected.

**TABLE 2:** Means, standard deviations, correlations, and alphas of the modeled latent variables.

**Variable**	**Mean**	***SD***	**1**	**2**	**3**	**4**	**5**	**6**
1. TFL	3.62	0.80	(0.91)					
2. PAL	2.19	0.77	–0.64	(0.88)				
3. RA	2.68	1.18	–0.46	0.36	(0.81)			
4. TCL	5.42	0.90	0.63	–0.53	–0.51	(0.85)		
5. Job Autonomy	5.88	0.97	0.29	–0.21	–0.25	0.35	(0.77)	
6. Anxiety	3.61	1.58	–0.34	0.35	0.41	–0.39	–0.15	(0.88)

**N* = 501. Cronbach’s alpha in parentheses. All correlations significant at the 0.01-level (two-tailed).*

### Confirmatory Factor Analysis

The measurement model comprised the six latent factors (TFL, PAL TCL, RA, job autonomy, anxiety) and their indicators. TCL was included as a latent variable with four indicators [mean scores of factors: (1) group interaction and group support, (2) goal alignment, (3) open exchange, and (4) regular contact] as the previously conducted CFA revealed.

The results of the measurement model revealed a reasonable fit with the data [χ^2^_(__881__)_ (*N* = 501) = 2364.798, *p* < 0.001; χ*^2^*/*df* = 2.684; TLI = 0.90; CFI = 0.92; RMSEA = 0.058]. All items loaded in the expected direction on their corresponding latent factors, and all standardized factor loadings were significant at *p* < 0.001, ranging between 0.481 and 0.915, supporting convergent validity. Moreover, all correlations were below 0.80, supporting discriminant validity. Therefore, the hypothesized factor structure of the measurements was supported.

### Assessment of Common Method Bias

To assess common method bias, we applied the single-common-method-factor approach (Podsakoff et al., 2012). The differences only ranged between −0.084 and 0.11, i.e., the effect sizes of the indicators on their theorized latent factor: (a) were not substantially different when controlling for common method variance and (b) were mostly inflated, but also deflated in some cases. Since all values scored lower than our cut-off criterion of <0.2, we did not impute the factor loadings of the model with single common method factor into our hypothesized structural equation models.

### Testing the Model for Transformational Leadership

We first tested the moderated mediation model where perceived job autonomy moderates the link between TFL and RA. The estimation of the proposed model yielded adequate fit indices [χ^2^_(__1258__)_ (*N* = 501) = 2791.93, *p* < 0.001; χ*^2^*/*df* = 2219.34; TLI = 0.91; CFI = 0.92; RMSEA = 0.047]. H1a and H2a were supported. TFL had a significant indirect effect on follower’s level on anxiety via TCL and RA. More specifically, the standardized indirect effect from TFL through RA and TCL to anxiety was −0.30 (95% CI [−0.46, −0.16]). As can be seen in Table 3, all mediating paths were significant. The direct path linking TFL to anxiety was not significant, therefore revealing that the relationship between TFL and anxiety is fully mediated by role RA and TCL.

**TABLE 3:** *B* coefficients, *SE*, *p*-values, and 95% B CI of the transformational leadership model (TFL × Autonomy moderation).

					**95% B CI**	
**Predictor**	**Outcome**	***B***	***SE***	***p***	**Lower**	**Upper**
TFL	TCL	0.50	0.06	^∗∗∗^	0.37	0.63
PAL	TCL	–0.19	0.06	^∗∗∗^	–0.33	–0.083
TFL	RA	–0.34	0.07	^∗∗∗^	–0.51	–0.19
PAL	RA	0.20	0.07	^∗∗^	0.054	0.36
Autonomy	RA	–0.15	0.05	^∗∗^	–0.29	–0.017
TFL × Autonomy	RA	0.19	0.05	^∗∗∗^	0.042	0.43
RA	ANX	0.50	0.09	^∗∗∗^	0.26	0.66
TCL	ANX	–0.28	0.11	^∗∗^	–0.53	–0.047
PAL	ANX	0.16	0.11	0.18	–0.072	0.38
TFL	ANX	–0.04	0.13	0.81	–0.31	0.25

**N* = 501. ^∗∗∗^ indicates a *p*-value <0.01. ^∗∗^ indicates a *p*-value <0.05. ANX = anxiety.*

**FIGURE 3:**
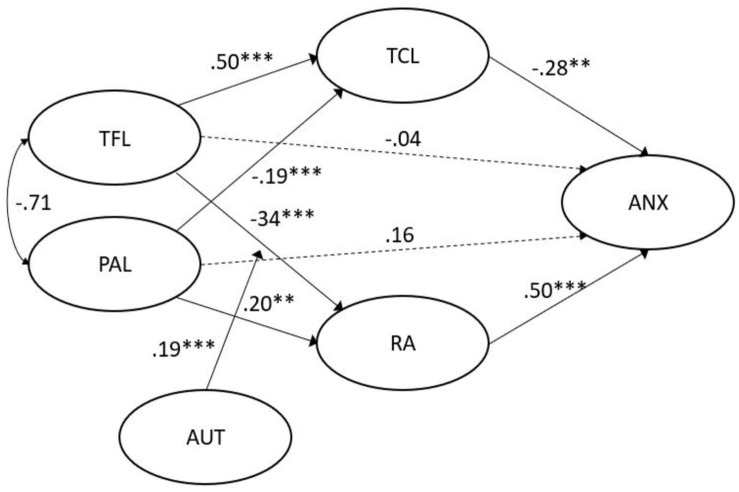
Empirical model TFL. ^∗∗∗^ indicates a *p*-value < 0.01. ^∗∗^ indicates a *p*-value < 0.05.

H3a was also supported: the model showed a positive significant interaction effect between TFL and job autonomy with 0.19 being significant at the 0.05-level and 95% CI = [0.042, 0.43]. The positive interaction shows: the higher the degree of job autonomy, the greater the effect of TFL on RA. Figure 3 shows the identified interactions.

### Testing the Hypothesized Model for Passive-Avoidant Leadership

We further tested the moderated mediation model where perceived job autonomy moderates the link between PAL and RA. The estimation of the proposed model yielded acceptable fit indices [χ^2^_(__1258__)_ (*N* = 501) = 2558.62, *p* < 0.001; χ*^2^*/*df* = 2.034; TLI = 0.93; CFI = 0.93; RMSEA = 0.05]. Hypotheses 1b and 2b were supported. More specifically, the standardized indirect effect from PAL through RA and TCL to anxiety was 0.15 (95% CI [0.064, 0.28]). The direct path linking PAL to anxiety was not significant, therefore revealing that the relationship between PAL and anxiety is fully mediated by RA and TCL (Table 4).

**TABLE 4:** *B* coefficients, *SE*, *p*-values, and 95% *B* CI of passive-avoidant leadership model (PAL × Autonomy moderation).

					**95% B CI**	
**Predictor**	**Outcome**	***B***	***SE***	***p***	**Lower**	**Upper**
PAL	TCL	–0.19	0.05	^∗∗∗^	–0.32	–0.074
TFL	TCL	0.50	0.07	^∗∗∗^	0.37	0.63
PAL	RA	0.22	0.07	^∗∗∗^	0.065	0.38
TFL	RA	–0.34	0.07	^∗∗∗^	–0.51	–0.19
Autonomy	RA	–0.15	0.05	^∗∗^	–0.29	–0.023
PAL × Autonomy	RA	–0.71	0.47	^∗∗^	–5.72	–0.077
RA	ANX	0.46	0.09	^∗∗∗^	0.27	0.65
TCL	ANX	–0.29	0.11	^∗∗^	–0.53	–0.056
TFL	ANX	–0.03	0.13	0.85	–0.31	0.26
PAL	ANX	0.17	0.11	0.17	–0.062	0.40

**N* = 501. ^∗∗∗^ indicates a *p*-value <0.01. ^∗∗^ indicates a *p*-value <0.05. ANX = anxiety.*

**FIGURE 4:**
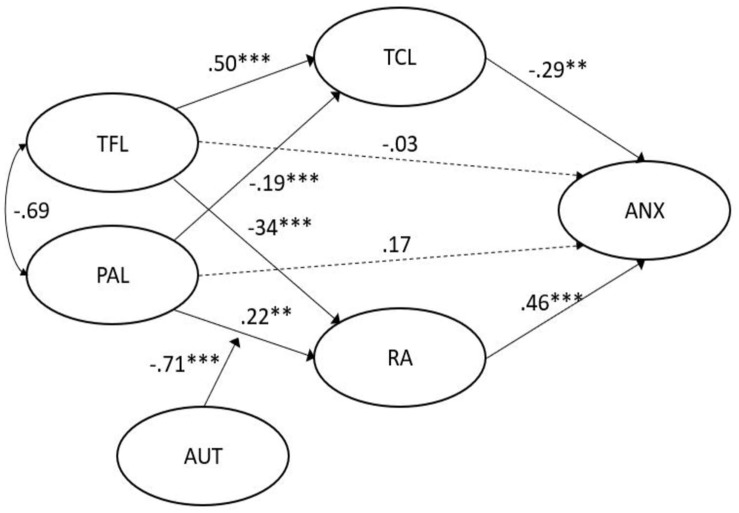
Empirical model PAL. ^∗∗∗^ indicates a *p*-value < 0.01. ^∗∗^ indicates a *p*-value < 0.05.

Hypothesis 3b was also supported, since there was a significant interaction effect between PAL and RA. The moderation effect was −0.71 (95% CI [−5.72; −0.077]). That is, perceived job autonomy had a buffering effect on the negative link between PAL and RA. Figure 4 illustrates the identified relationships.

## Discussion

### Findings

This research made four contributions to leadership research. First, it addressed the limitations of previous research on TFL and employee well-being by bringing together job demands and job resources to explain how positive and negative leadership influences follower well-being. Second, it expanded the complexity of this research field’s commonly used mediator models by modeling moderated mediation models, thereby finding additional answers to when leadership behaviors influence job demands. Third, it emphasized the importance of bringing together resources and demands from motivational and relational and social-cognitive categories when analyzing well-being at the workplace. Fourth, TFL was analyzed in contrast to PAL to better understand the role of the positive TFL.

The principal aims of the current study were to examine how and when transformational leaders may contribute in promoting their followers’ well-being, and what happens when leaders show opposing behaviors. Based on the JD-R model, two moderated multiple mediation models were hypothesized where job demands and job resources mediate the relationship between TFL – respectively, PAL – and anxiety, and where job autonomy as an important job characteristic determines the quality of the leadership–job demand relationship.

The main findings are that the relationships between TFL and PAL on the one hand and anxiety on the other hand are fully mediated by the motivational RA (Hypothesis 1) and the relational and social-cognitive TCL (Hypothesis 2) paths, confirming our two hypotheses regarding the question how leadership influences employee well-being. In line with previous research, the health-impairment process via the job demand was stronger than the link via job resource on anxiety (Schaufeli and Taris, 2014; Schaufeli, 2015). TFL’s relationship with TCL was stronger than its relationship with RA. In contrast, PAL’s relationship with RA was higher than its relationship with TCL. This is in line with previous research, that suggested that positive leadership indirectly affects follower’s well-being by primarily increasing resources, whereas negative leadership does so by primarily impacting job demands (Schaufeli, 2015; Inceoglu et al., 2018). The results suggest that transformational leaders have a slightly stronger effect via relational social-cognitive than motivational paths, but motivational paths are closer linked to anxiety. Specifically, transformational leaders may reduce employee anxiety by fostering a positive TCL. Such a climate may be induced by promoting open exchange, knowledge sharing, mutual trust, communication, and goal setting between team members. Another mechanism through which transformational leaders may contribute to the reduction of their followers’ anxiety is by giving them guidance for efforts, by encouraging their self-confidence to pursue new pathways for growth, and by reducing ambiguity (Harms et al., 2017). The absence of a direct effect of TFL on employee anxiety is noteworthy and in line with existing research that has demonstrated that the relationship between TFL and well-being is a fully mediated relationship (e.g., Arnold et al., 2007; Tafvelin et al., 2011; Kelloway et al., 2012). In turn, the results showed that PAL not only increases motivation-related job demands but also decreases important job resources (relational and social-cognitive TCL) and is thus by far not a form of zero-sum leadership, but a severe factor for employee stress. This is in line with previous research (Skogstad et al., 2007; Hetland et al., 2011).

Another finding is that job autonomy acts as a moderator for the relationship between TFL and RA. Our results suggest that TFL is generally able to decrease the demand of RA, but that TFL works better when the situation provides the employee enough freedom to act. In other words, when job design constraints prevail, positive leadership is less effective to reduce job demands, which is in line with previous research (Volmer et al., 2012).

In contrast to this amplifying moderating effect and in line with previous research (Hetland et al., 2011), job autonomy buffered the connection between PAL and RA, suggesting as it is to be an impactful resource when dealing with leader passivity. Furthermore, the considerably high negative covariation between TFL and PAL strengthened the view of PAL as a direct antagonist of TFL.

### Theoretical Implications

Apart from the described findings, the study further revealed five theoretical implications. We expanded the ongoing research work on the JD-R model and leadership both conceptually and methodologically. First, conceptually, we included job resources and job demands, on both motivational and relational and social-cognitive level. To our knowledge, no research up until now has examined TCL and RA as possible mediators between the TFL well-being relationship. Specifically, although there is plenty of evidence that transformational leaders increase job resources (e.g., Arnold et al., 2007; Nielsen et al., 2008) and in turn promote employee well-being, only a small amount of research has proposed models in which transformational leaders exert their health promoting effects by reducing job demands (Hentrich et al., 2017). Second, methodologically, we modeled not only mediator or moderator models, but created moderated multiple mediation models, thereby integrating different interactions into one frame. In doing so, we followed the call of previous meta-analyses to combine moderators and mediators in the same model (Arnold, 2017).

Third, this research could highlight the importance of positive leadership for follower’s well-being by contrasting the effects of TFL and PAL, and also the interplay of resources (TFL and job autonomy). Fourth, TFL and PAL were not directly associated with follower’s well-being, suggesting full indirect relationships.

### Practical Implications

With the aim of maintaining a mentally healthy workforce, leadership training may be a productive occupational health intervention. Leaders should be aware of their potentially significant role in helping employees cope with anxiety – and that passivity can have severe consequences – and so be trained to that they can adopt more transformational behaviors.

Training programs may be designed not only for leaders but for teams as well. Employees need to be aware that their team may consist of a valuable resource when it comes to learning, dealing with ambiguity and, most importantly, their well-being. Therefore, training programs on motivational, relational, and social-cognitive aspects that promote communication within the team, knowledge transfer, and planning skills may result effective. Moreover, through team building activities, employees may build trust with their fellow team members and improve their communication.

Ultimately, and next to HR practices, job design measures that grant employees more autonomy in the way they carry out their tasks should be considered.

### Limitations and Future Research

Although our study contributes to the further understanding of the relationship between TFL/PAL and employee well-being, it has certain limitations. First, we applied a cross-sectional design, which clearly limits the degree to which we could make causal inferences regarding the relationships proposed between the variables. However, following Hetland et al. (2011), only experiments overcome cause and effect issues. For the purpose of this manuscript, studying the phenomena linked to a real-life context seemed appropriate. Consistent with Cerin’s (2010) recommendations, we mitigated the limitations associated with cross-sectional mediation analysis by relying on a large sample, which helps reduce bias in regression estimates due to measurement error (Kline, 2016).

Additionally, the proposed models are supported by previous research and current theory, while the relationship between TFL and well-being has also been demonstrated by longitudinal studies (e.g., Tafvelin et al., 2011). Furthermore, the JD-R model has been attested longitudinal evidence in reviews, which is why other research regarding this relationship in the context of the JD-R model has also applied cross-sectional designs (e.g., Schaufeli, 2015; Hentrich et al., 2017). Explicitly, Schaufeli and Taris (2014) state in their critical review of the JD-R model that “job demand and job resources have an impact over time on burnout” (Schaufeli and Taris, 2014, p.48). In any case, the findings of the present study would need to be replicated by longitudinal or experimental designs, so that the mediation effects causality can be adequately tested.

Second, our study used only one source of information for data gathering (self-reported questionnaire), which raises concerns about common method bias (Podsakoff et al., 2003). Hence, estimates of the effects of the predictors on the criterion variable and other constructs might be artificially inflated, deflated, or non-significant (Podsakoff et al., 2012). To mitigate this effect, we followed state of the art recommendations and applied procedural (e.g., avoiding ambiguous items by using simple and concise statements and syntax, reverse coding, and counterbalancing question order) and statistical remedies (single-common-method-factor approach) to statistically control for a common latent variable (Podsakoff et al., 2012). Although we designed our questionnaire carefully and statistical testing suggested that common method bias was not an issue in our study, we encourage future researchers to collect data from multiple sources in order to reduce Type-I error probability (Podsakoff et al., 2012).

Third, we built our research study on the JDR as it is the most prominent and broad framework that helps explaining the indirect relationship between TFL and well-being. Other approaches, e.g., leader–member exchange theory (Graen and Uhl-Bien, 1995), can add further information and should be used in future research.

Fourth, we included only one job resource and job demand as mediators, and the list of potential mediators is unquestionably longer than this (see Arnold, 2017 for a meta-analysis on mediators and moderators). When it comes to role stressors, due to the particularities of working in a research institution, the present study only included RA as a possible mediator. Therefore, future research should examine the possible mediating effects of role conflict and role overload, for instance. Regarding the detrimental effect of leader passivity, future research should further investigate which resources work for which particularly increased job demand, since it is likely that only specific resources work for specific demands (Schaufeli and Taris, 2014).

Fifth, the interest of the present study focused on individual health. However, future studies should consider analysis at a group level and examine whether TFL may affect followers’ collective well-being.

Sixth, the sample was unbalanced toward researchers and male participants, which was due to the true distribution of genders (34% were women) and jobs (55% were researchers) in the organization (based on HR data from the year 2014). However, our data correctly represent today’s R&D sector with its limited gender diversity. Future research with more balanced samples would be interesting to confirm or refute the obtained results.

## Conclusion

In summary, leadership behavior affects the well-being level, namely, anxiety, of employees by influencing perceived demands and resources to cope with demands. TFL is not only associated with a positive TCL, but also with reducing RA. TFL works better in high-autonomous contexts. In contrast, leader passivity increases job demands and reduces job resources. Autonomy-promoting job designs buffer the link between PAL and RA.

## Data Availability Statement

The datasets generated for this study are available on request to the corresponding author.

## Ethics Statement

The studies involving human participants were reviewed and approved by two ethics committees: by the workers’ council (German: “Gesamtbetriebsrat”) of the participating organization, and by the academic commission of the Ph.D. program HDK 14 (“Comissió de Acadèmica del programa de Doctorat HDK 14 - Psicologia Social i de les Organitzacions”) of the Universitat de Barcelona (UB). The patients/participants provided their written informed consent to participate in this study.

## Author Contributions

RB, JC, and DL contributed to definition of research objectives, models, and hypotheses. RB and J-PL contributed to provision of materials (i.e., questionnaires). J-PL contributed to data collection. RB and DL contributed to data analysis plan. JC and DL contributed to data analysis. RB and JC contributed to principal article writing. RB, DL, and J-PL contributed to article revision and proofreading. RB, DL, JC, and J-PL contributed to final approval.

## Conflict of Interest

The authors declare that the research was conducted in the absence of any commercial or financial relationships that could be construed as a potential conflict of interest.
